# A prospective examination of mask anxiety during radiotherapy for head and neck cancer and patient perceptions of management strategies

**DOI:** 10.1002/jmrs.346

**Published:** 2019-07-25

**Authors:** Jodie L. Nixon, Bena Brown, Amanda E. Pigott, Jane Turner, Elizabeth Brown, Anne Bernard, Laurelie R. Wall, Elizabeth C. Ward, Sandro V. Porceddu

**Affiliations:** ^1^ Occupational Therapy Department Princess Alexandra Hospital Brisbane Australia; ^2^ School of Health and Rehabilitation Sciences The University of Queensland Brisbane Australia; ^3^ Speech Pathology Department Princess Alexandra Hospital Brisbane Australia; ^4^ Centre for Functioning and Health Research Metro South Hospital and Health Service Woolloongabba Australia; ^5^ Faculty of Medicine The University of Queensland Brisbane Australia; ^6^ Radiation Oncology Department Princess Alexandra Hospital Brisbane Australia; ^7^ QFAB Bioinformatics, Institute for Molecular Bioscience, The University of Queensland Brisbane Australia

**Keywords:** Head and neck cancer, mask anxiety, radiotherapy, shell, thermoplastic mask

## Abstract

**Introduction:**

Distress related to wearing an immobilisation mask for radiotherapy treatment (RT) is a common experience for the person undergoing RT for head and neck cancer (HNC). Described as ‘mask anxiety’, there is little known about the patterns of this distress through the course of the treatment or what strategies are being used by people to help alleviate mask anxiety.

**Methods:**

The study used a prospective cohort design to examine the patterns of patient–reported mask anxiety during the course of RT, using a modified Distress Thermometer (DT) and a survey to explore strategies patients used to assist their mask anxiety.

**Results:**

Thirty‐five participants, who identified as experiencing mask anxiety, were followed throughout RT treatment. At baseline, females were more likely to experience higher mask anxiety (*P* = 0.03). Across the course of treatment, mask anxiety significantly (*P* < 0.001) reduced within the total cohort. In 72% of participants, the level of initial distress was found to reduce over time. Only 22% experienced mask anxiety that remained constant. Few (6%) experienced an increase in mask anxiety across the course of RT. Participants reported relying on intervention from health professionals, self–taught strategies, music, visualisation and medication to manage their mask anxiety.

**Conclusions:**

Due to its high prevalence and variable patterns over time, it is recommended that routine screening for mask anxiety be implemented as standard care throughout the course of RT for HNC. Multiple, diverse strategies are being used by patients and studies are needed to develop effective interventions for managing mask anxiety.

## Introduction

People diagnosed with head and neck cancer (HNC) are at a high risk of experiencing distress along the continuum of cancer treatment.^1^ Distress is a term that has been adopted to describe an unpleasant experience of a psychological, social, spiritual and/or physical nature.^2^ In the population with HNC, patient–reported distress before and after radiotherapy (RT) is prognostic for poorer survival,^3^ with long–term challenges associated with physical functioning, fatigue and reduced social supports contributing to distress.^1^, ^3^, ^4^, ^5^, ^6^ Furthermore, distress in the population with HNC has been found to be related to the use of the thermoplastic mask required to immobilise patients to ensure precision of RT treatment.^7^, ^8^, ^9^

Early studies which have examined distress and claustrophobia associated with the use of the thermoplastic mask have reported prevalence rates between 14% and 58%.^9^, ^10^, ^11^ This experience has been termed ‘mask anxiety’ and encompasses feelings of distress, anxiety and claustrophobia associated with fitting and wearing the mask for RT. The causes of mask anxiety appear multifactorial. Studies have found personal factors such as taking psychoactive medication, reported vulnerability associated with claustrophobia, fear of movement restriction, concurrent stressors and a history of anxiety attacks as contributors to disrupted pre‐treatment computerised tomography (CT) simulation (i.e. radiotherapy treatment planning session) as potential contributors to the phenomenon of mask anxiety.^7^, ^8^, ^9^

Studies to date have reported on the prevalence of mask anxiety at single time points,^9^, ^10^, ^11^ therefore little is known regarding the course and pattern of mask anxiety during RT. Previous studies have asked participants to describe the experience of wearing the mask and have found a combination of psychological (e.g. anxiety, distress, feelings of vulnerability, uncertainty, fear) and physiological (e.g. tachycardia, sweating, tachypnoea) responses to the use of the mask.^7^, ^9^ Another study reviewed the process of RT and presented case studies on anxiety related to the need for mask immobilisation which can potentially be treatment‐limiting.^12^ However to date there are no reports on whether these experiences reduce with familiarity of use with the mask or whether the experiences are ongoing through the treatment.

Mask anxiety has been reported to disrupt the first day of RT for 24% of people (*n* = 90)^7^ and in one study it has been described by participants as one of the worst things about RT.^13^ Participants have proposed practical strategies (e.g. removing parts of the mask to aid vision/breathing), pharmacological and psychological interventions (e.g. visualisation and mindfulness) to optimise their comfort during the process. Limiting this evidence is that these strategies have been suggested by participants prior to their first session of RT treatment^9^ or by a small number of people who did not actually experience mask anxiety.^14^ Pilot studies which have explored possible interventions have found early positive results for music therapy and mindfulness, however, similarly the participants within these studies were not specifically reporting mask anxiety.^15^, ^16^ Whilst the experience of mask anxiety is better recognised as a factor impacting on RT treatment for people with HNC, little is known about management strategies to minimise mask anxiety.

Mask anxiety is a common phenomenon, yet currently little is understood about its presentation over the course of RT treatment. There is also minimal information available to support effective interventions to assist patients to manage their anxiety symptoms. Hence the aims of this study were: (1) to determine the severity and course of mask anxiety during RT for HNC, and (2) identify patient–reported management strategies to minimise mask anxiety during RT for HNC.

## Methods

### Study design

This prospective cohort study examined the severity, course and self–identified management strategies of people who experienced mask anxiety during RT for HNC. This study was conducted at a single, quaternary hospital in Brisbane, Australia. Ethical clearance was received from the Metro South Hospital and Health Service Human Ethics Committee (HREC/13/QPAH437). All participants provided informed written consent.

### Participants

A consecutive sample of 250 participants was potentially eligible for recruitment from a time–limited sample between June 2015 and May 2017. Eligible participants were those who were: receiving definitive or post‐operative radiotherapy (PORT) or concurrent chemoradiotherapy (CXRT)for HNC, required a thermoplastic mask; their self–reported score was four or greater on the modified Distress Thermometer (DT) for mask anxiety described below; or they were referred by staff in the radiation unit as identifying as anxious about use of the mask.^2^, ^17^ The DT for mask anxiety was recorded as part of routine departmental screening at the time of CT simulation and then weekly during RT or participants were directly identified as having mask anxiety by the treating team during CT simulation. Exclusion criteria included: severe cognitive limitations; non‐English speaking; or significant vision, hearing or physical dexterity impairments which limited completion of the departmental, web–based screening tool, used throughout this study.

### Procedure

Once participants were identified and recruited to the study, they continued to complete the web–based departmental screening tool on a weekly basis, which included the modified DT for mask anxiety.^5^ All participants were immobilised with a perforated 5‐point thermoplastic mask that encompassed the head, neck and shoulders. The standard of care for any patient identified within the service as having mask anxiety involves a multidisciplinary team (MDT) approach that consists of: medical and nursing support for education and medication options; radiation therapy for education, offer of music and review of alternate positioning options such as mask modification and; occupational therapy for education, support and exploration of relaxation strategies. No additional training was given to staff. All participants recruited to this study were referred to these services and could participate in any strategy that the individual chose/preferred.

### Measures

As no validated tool existed to screen or measure mask anxiety at the time of project conceptualisation, a modified version of the DT was utilised for the current study.^9^ The DT is a widely used tool which rates patient–reported distress using a visual analogue scale. It was modified to specifically address patient‐experience of distress related to wearing the mask during RT for HNC: ‘Please circle the number (0–10) that best describes the level of distress you feel about wearing the thermoplastic mask for radiotherapy?’ A cut off score of 4 or above was considered clinically significant and a trigger for referral, as is consistent with other uses of the DT.^2^, ^9^, ^17^

At RT treatment completion, participants completed a one‐off survey to identify participant perceptions of management strategies offered to them and to what extent they felt these reduced their mask anxiety during treatment. The survey comprised ten items (Table 4) which were related to the available services at the study centre, as well as personal factors that may have impacted on their ability to tolerate the mask. Participants used a Likert scale to indicate how much each assisted their adjustment to wearing the mask (1 ‘strongly disagree’ to 5 ‘strongly agree’). For each question, a free–text section allowed participants to add comments and a final open–ended question allowed patients to make additional comments or suggestions regarding their experience.

### Data analysis

Descriptive statistics were conducted for continuous data using mean and standard deviation (SD) or median and interquartile range (IQR) when data were not normally distributed. Normality of the data was assessed using Shapiro‐Wilk test. Categorical variables were reported as frequency and percentage.

Data from weekly time points were collapsed into three main time points to investigate mask anxiety during RT: baseline (either CT simulation or week 1 of treatment in the absence CT simulation data); mid‐RT (week 2 of treatment for shorter course RT; or week 4 of treatment for 6/7‐week RT) and; end of RT (week 4 for shorter course or week 6/7 weeks, dependent on treatment length).

Relationship between mask anxiety at baseline and demographic variables, disease stage and treatment received was assessed using a *t*‐test (gender, surgery) or ANOVA (comparison of treatment type CXRT vs. PORT vs. RT).

To assess the course of mask anxiety over time, a linear mixed model was performed with time as fixed effect and participant as random effect (random intercept). Tukey’s HSD post‐hoc test with multiple testing adjustments, using Bonferroni correction was performed for pairwise comparisons and adjusted mean differences due to the outliers, standard errors and adjusted *P*‐values are reported. All analyses were performed using the R statistical software and *P*‐values were two‐tailed with *P* < 0.05 considered statistically significant.^18^

## Results

Of the 250 participants treated for HNC during the study time frame, 35 participants met all eligibility criteria and consented to participate. The majority of participants were male (*n *= 26, 74%), with a median age of 63 years (range 30–83 years), being treated for oral cavity/oropharyngeal cancers using surgery and post‐operative radiotherapy or concurrent chemoradiotherapy (Table 1). Data from 32 participants was available across the three time points for mask anxiety. All participants completed the survey. No participants required re‐CT or a new mask fitted during their RT, however four participants had shim changes to accommodate weight and swelling changes.

**Table 1:** Demographics

Demographic variable	No of participants (*n* = 35) *n* (%)
Age (Median, IQR)	63 (56, 70)
Gender	
Male	26 (74.3%)
Female	9 (25.7%)
Treatment	
PORT	17 (48.6%)
CRT	13 (37.1%)
RT	5 (14.3%)
No. of fractions RT (Median, IQR)	30 (30, 35)
Chemotherapy/systemic therapy	
No	22 (62.9%)
Cetuximab	4 (11.4%)
Cisplatin	9 (25.7%)
Surgery	
No	14 (40%)
Yes	21 (60%)

PORT, post–operative radiotherapy; CRT, chemoradiotherapy; RT, radiotherapy; IQR, interquartile range.

Demographic variables of age or treatment type were not significantly associated with mask anxiety severity at baseline (Table 2). From this group of participants, mask anxiety at baseline was significantly higher in females (M = 7.25, SD 2.71) than males (M = 4.48, SD 2.79) (*P* = 0.03).

**Table 2:** Mask anxiety level at baseline by demographic group

	*n*	Mean (±SD)	*P*‐value	*t* (df)	F (df)
Gender					
Male	26	4.48 (±2.79)	0.03	2.50 (12.12)	–
Female	9	7.25 (±2.71)			
Treatment					
PORT	15	4.8 (±2.81)	0.18	–	1.76 (30)
CRT	13	4.69 (±3.3)			
RT	5	7.4 (±1.82)			
Chemotherapy/systemic therapy					
No	20	5.45 (±2.8)	0.50	0.68 (22.71)	–
Yes	13	4.69 (±3.3)			
Surgery					
No	14	5.21 (±3.42)	0.92	0.10 (24.04)	–
Yes	19	5.11 (±2.71)			

SD, standard deviation; PORT, post–operative radiotherapy; CRT, chemoradiotherapy; RT, radiotherapy.

The severity of mask anxiety over time changed. Linear mixed modelling revealed a significant difference in mask anxiety over time (*P* < 0.001), with significant improvement from mid‐RT to end‐RT (*P* < 0.001) and from baseline to end‐RT (*P* < 0.001), with a trend for improvement from baseline to mid‐RT (*P* = 0.06) (Table 3).

**Table 3:** Modified distress thermometer rating across time points

	Mean (±SD)	Comparison Adj. mean difference (±SE)	Adjusted *P*‐value
Baseline (*n* = 33)	5.15 (±2.98)	versus mid‐point 1.19 (0.53)	0.06
Mid‐point (*n* = 32)	3.94 (±2.34)	versus end‐point 1.84 (0.54)	<0.001
End‐point (*n* = 32)	2.09 (±2.61)	versus baseline −3.03 (0.53)	<0.001

SD, standard deviation; SE, standard error.

**Figure 1:**
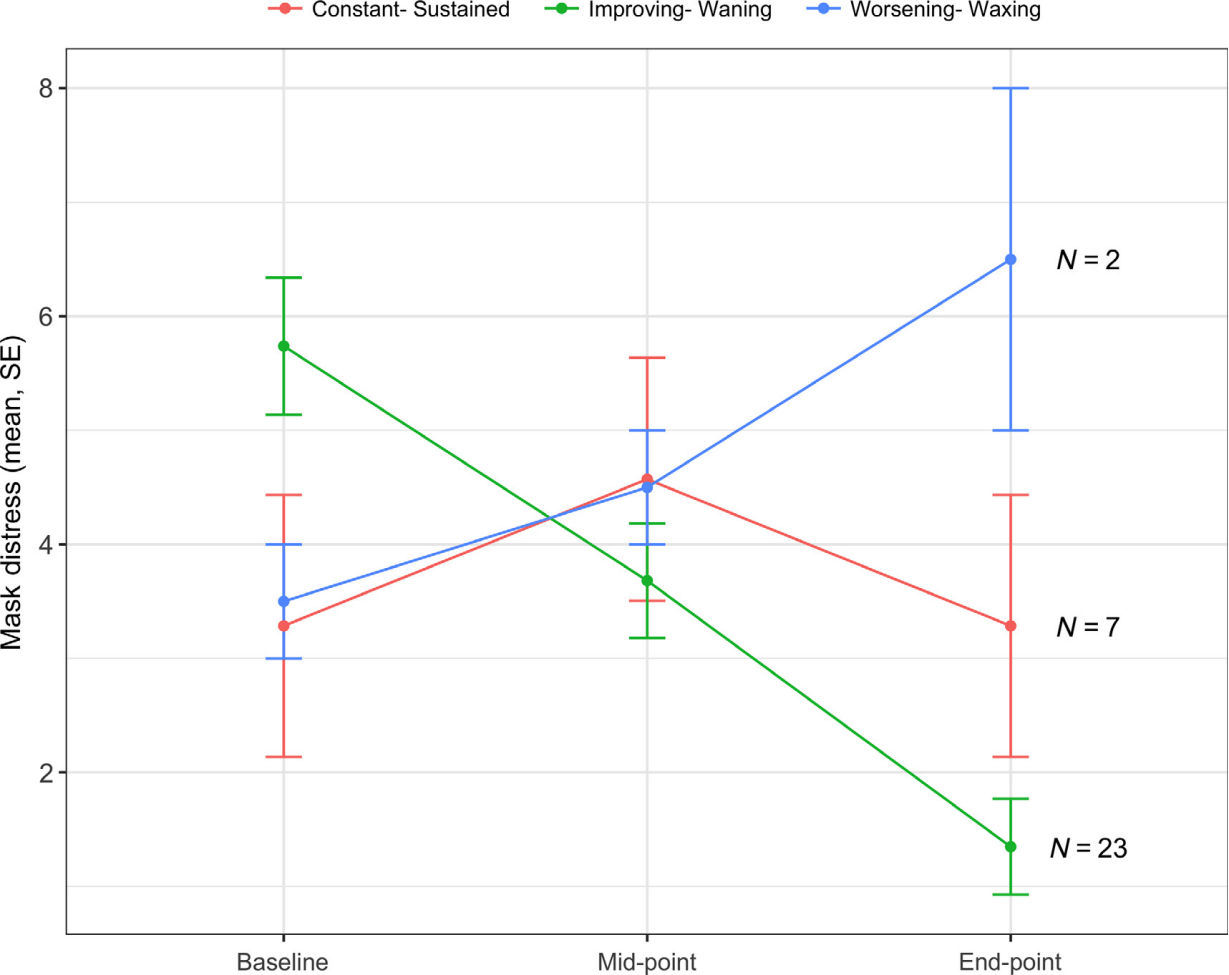
Patterns of mask anxiety

Although an overall group pattern of reduced anxiety levels was observed for the total cohort, individual analysis revealed the severity of mask anxiety reduced from baseline to end‐RT for 23 participants (72%), with a median decrease of six points on the DT. Mask anxiety severity remained stable for seven (22%) and increased for two (6%), with a median increase of three points on the DT. Mask anxiety was reported by 22% of the participants at every occasion during RT (Fig. 1).

The survey data revealed that 97% of participants understood the importance of wearing the mask during RT for HNC. Over the course of their RT, 63% reported they ‘got used to wearing the mask’. Of the participant–identified strategies to minimise mask anxiety, the majority of participants agreed that discussions with health professionals were helpful in minimising mask anxiety (occupational therapy 86%, radiation therapy 86%, nurses 83% and doctors 76%) (Table 4). Self–taught strategies (including breath regulation and meditation) and listening to music during RT were reportedly helpful for 84% and 72% participants respectively, with visualisation techniques less commonly reported as helpful (49%) (Table 4). Of those participants who were prescribed medication to minimise mask anxiety (*n* = 15, 43%), 60% reported it was helpful, however 40% discontinued medication after a few days as they no longer required it or felt the benefit was not justified given the side effects or activity restriction (e.g. driving) related to the medication. Free–text and open–ended data were reported by 24 participants. These participants reported staff communication during RT (*n* = 14), as well as practical adjustments to the mask (i.e. removing the material around the eyes/mouth) (*n* = 10) were helpful in minimising mask anxiety.

**Table 4:** Post‐treatment survey: participant perceptions of reducing distress associated with mask anxiety

Concept	Disagree *n* (%)	Neutral *n* (%)	Agree *n* (%)	N/A *n* (%)
Understood importance of wearing mask	1(3)	0(0)	34(97)	0(0)
Discussions with doctor were helpful	8(22)	1(3)	25(72)	1(3)
Discussions with nurse were helpful	2(6)	4(11)	29(83)	0(0)
Discussions with RT were helpful	2(6)	3(8)	30(86)	0(0)
Discussions with OT were helpful	1(3)	3(8)	30(86)	1(3)
I used self–taught strategies	1(3)	1(3)	33(94)	0(0)
Visualisation was helpful	4(11)	5(14)	17(49)	9(26)
Medication was helpful	3(8)	3(8)	9(26)	20(58)
Music was helpful	1(3)	2(6)	25(72)	7(20)
I got used to wearing the mask	5(14)	6(17)	22(63)	2(6)

RT, radiation therapist; OT, occupational therapist.

## Discussion

Given the negative survival sequelae associated with distress in the HNC population^3^ and that mask anxiety is a well–identified contributor to distress, it is essential for patient care that cancer centres have an understanding of mask anxiety with the HNC population receiving RT.^9^ Our study is first to report the longitudinal severity and course of mask anxiety during RT for HNC. Firstly, females from this sample were more likely to have higher average scores of mask anxiety than males, with no other demographic variables evident. A number of studies of distress in cancer populations have found distress higher in females than males,^19^, ^20^ however, no previous studies have reported gender as a precipitant to mask anxiety and as such warrants further exploration.

Participants were aware of the importance of the mask and various management strategies were identified by participants as helping to manage mask anxiety, however in the survey data more than one third of participants reported they did not get used to wearing the mask throughout RT. To date, no other study has reported on the course of mask anxiety through the RT treatment. One other study interviewed patients (*n* = 241) weekly through the course of treatment regarding the tolerability of the mask, however only reported the result at one time point.^11^ Our study identified three patterns of mask anxiety through the course of RT treatment: (1) waning (decreasing severity over time), (2) sustained (no change to mask anxiety severity over time) and (3) waxing (increasing severity of mask anxiety over time). The current study did not examine potential causative factors between management strategies and patterns in mask anxiety. Further studies should assess if strategies employed by patients are effective in reducing the experience of mask anxiety. Possible hypotheses for lack of improvement in the experience of mask anxiety may be pre‐existing claustrophobia not amenable to the strategies discussed here or an increase in side effects associated with RT/chemoradiation treatment that would impact on tolerance of the mask (e.g. pain, thick oropharyngeal secretions, oedema). As the current study failed to examine such issues alongside the mask anxiety patterns it is impossible to make any definitive statements to explain the different patterns observed in the cohort. Further research into these possible influencing factors is needed to help predict outcomes for individuals. The data highlights that mask anxiety changes over the course of RT and should be screened at regular intervals alongside other factors known to cause distress, to support the people who experience these side effects.

The results of the current study also suggest that patients who require a mask for RT should have access to health professional intervention to minimise their experience of mask anxiety and offer potential options to support their completion of treatment. One fast‐acting intervention for mask anxiety is the use of medication.^7^ Antianxiety drugs known as benzodiazepines are commonly used for this purpose (e.g. Lorazepam), which act on the central nervous system to relieve nervousness.^21^ The dose is prescribed by the treating doctor, alleviating symptoms of nervousness after 30–60 min and lasting up to four hours. Of our cohort, less than half the group were prescribed medication and only 25% of the total cohort found medication helpful to minimise their mask anxiety, with reports of side effects, the need for extra medication and restrictions to their activities (i.e. being unable to drive) consistent with previous reports.^9^ Although medication may minimise mask anxiety in a proportion of patients, the associated complications identified in the current study support the consideration of alternative options for patients with HNC during RT.

The majority of participants (86%) rated discussions with health professionals as helpful in reducing mask anxiety. In the study centre, the MDT provided standard care interventions for patients who experience mask anxiety. The current study showed that participants reported all health professional interactions as helpful (including radiation therapist, occupational therapy, nursing and medical staff), indicating potential multidisciplinary involvement to screen for and discuss mask anxiety with patients who require a mask for RT. Intervention and support for mask anxiety could be delivered by any health professional discipline, however as suggested by participants in other studies it is essential that this health professional intervention is underpinned by communication skills to ensure the choice of words and responses are effective in promoting an effective response.^22^, ^23^, ^24^

The use of music during RT, self–taught strategies and visualisation were described in the current study as helpful in minimising mask anxiety during RT for HNC. Rossetti et  al.^15^ found that patients with HNC had a significant reduction in distress level when a 30‐min consultation with a music therapist was provided prior to RT simulation, compared to a control group. These sessions involved participants selecting music that was meaningful for them to be played during the procedure.^15^ Whilst not utilising a formal music therapy intervention, the study centre routinely offers patient–selected music to be played during RT delivery. The current study supports the use of music with 72% of participants reporting music during RT was helpful for minimising mask anxiety. Additionally, a pilot study of 19 HNC participants used a tailored mindfulness intervention and found it to be feasible and acceptable to minimise general distress (not specific to mask anxiety) before physical symptoms became burdensome.^16^ The current study also found participant self–taught techniques such as breath regulation, meditation and visualisation (common components of mindfulness) were helpful for up to 84% in reducing their experience of mask anxiety. With strategies such as these being identified as beneficial for alleviating general distress and mask anxiety, future studies should focus on the investigation of alternative intervention models to best support patients experiencing mask anxiety.

### Limitations

There are a number of limitations to this study. The current study recruited small numbers potentially due to recruitment occurring through standard care verses a targeted research and as such disease stage could not be analysed due to range of stages as a contributing factor to mask anxiety.

Future studies recruiting a larger cohort should aim to verify the waxing/waning/sustained hypothesis of mask anxiety patterns. The survey utilised in the current study was designed to explore the use of supports currently available in the study centre and evidence reported in the literature at the time of study design. As such, novel intervention methods that have since been developed and investigated were not included. Future studies are recommended to investigate multidimensional interventions to determine effectiveness in reducing mask anxiety.

## Conclusions

Three patterns of mask anxiety for the group undergoing RT for HNC have been proposed: waxing, waning and sustained. Participants identified a number of interventions that helped minimise mask anxiety include health professional intervention, self–taught strategies (breath regulation and meditation), music, visualisation and the use of medication. Future studies are recommended to employ larger cohorts to describe the patterns of mask anxiety and to investigate interventional strategies.

## Conflict of Interest

The authors declare no conflict of interest.
